# Omicron BA.5 Neutralization among Vaccine-Boosted Persons with Prior Omicron BA.1/BA.2 Infections

**DOI:** 10.3201/eid2812.221304

**Published:** 2022-12

**Authors:** Rune M. Pedersen, Line L. Bang, Ditte S. Tornby, Lone W. Madsen, Dorte K. Holm, Thomas V. Sydenham, Isik S. Johansen, Thøger G. Jensen, Ulrik S. Justesen, Thomas E. Andersen

**Affiliations:** University of Southern Denmark, Odense, Denmark

**Keywords:** COVID-19, respiratory infections, severe acute respiratory syndrome coronavirus 2, SARS-CoV-2, SARS, coronavirus disease, zoonoses, viruses, coronavirus, Omicron, BA.1, BA.2, BA.5, vaccines

## Abstract

Worldwide, millions of persons have received multiple COVID-19 vaccinations and subsequently recovered from SARS-CoV-2 Omicron breakthrough infections. In 2 small, matched cohorts (n = 12, n = 24) in Denmark, we found Omicron BA.1/BA.2 breakthrough infection after 3-dose BNT162b2 vaccination provided improved Omicron BA.5 neutralization over 3-dose vaccination alone.

The SARS-CoV-2 Omicron variant has dominated the COVID-19 pandemic since late 2021, bringing >3 waves of increasingly immunity-evasive Omicron subvariants. An early wave of the Omicron subvariant BA.1 was rapidly replaced by a subvariant BA.2 wave during spring 2022. BA.2 was recently replaced by the even more transmissible BA.5, which is now globally dominant, accounting for 75%–95% of cases in most countries by August 2022 (*1*).

Because a large percentage of the worldwide population was infected by Omicron during early 2022, discussions have centered around whether convalescent patients acquired natural immunity that might later protect against BA.4/BA.5 subvariants (*2*). Serum neutralization studies have indicated that neither vaccination nor previous infection during the early Omicron waves offer effective protection against BA.5 (*3*–*6*). However, a recent large-scale epidemiologic study found that very few BA.5-infected persons had prior Omicron infection, indicating that previous Omicron infection might confer protection against BA.5 (*7*). To assess whether Omicron BA.1/BA.2 infection provides additional BA.5-specific neutralization capacity than vaccines alone, we compared authentic virus neutralization capacity among persons receiving 3-dose BNT162b2 vaccine (Pfizer-BioNTech, https://www.pfizer.com) regimens only and vaccinated persons who subsequently were infected with BA.1/BA.2.

We recruited healthy participants from among Odense University Hospital staff and the public in Odense, Denmark; all participants signed informed consent. We collected serum from the 24 participants in the vaccinated cohort during November 18, 2021–February 4, 2022, four weeks after they received the third BNT162b2 vaccination (Table). We collected serum from the 12 participants in the convalescent cohort during January 26–April 19, 2022, four weeks after Omicron BA.1/BA.2 breakthrough infection (Table). We performed plaque reduction neutralization tests (PRNTs) against authentic SARS-CoV-2 clinical isolates of Delta and Omicron BA.1, BA.2, and BA.5 variants, as previously described (*8*). We recorded PRNT_90_ titers, the highest dilution of a serum sample yielding >90% plaque reduction. We identified lineages by nanopore whole-genome sequencing using a MinION sequencing instrument (Oxford Nanopore Technologies, https://nanoporetech.com) and uploaded sequences to GenBank (accession no. ON055856 for Delta, ON055874 for BA.1, ON055857 for BA.2, and OP225643 for BA.5). We analyzed all serum samples for spike-specific antibodies by using the Liaison TrimericS IgG Quantitative Immunoassay (DiaSorin, https://www.diasorin.com). To verify SARS-CoV-2–naive status among the vaccinated cohort, we analyzed serum for nucleocapsid-specific antibodies by using Alinity SARS-CoV-2 IgG assay (Abbott Diagnostics, https://www.abbott.com). This study was approved by the Regional Committees on Health Research Ethics for Southern Denmark (approval no. S 20210007C). Experiments involving live SARS-CoV-2 virus were conducted in Biosafety Level 3 facilities (license no. 20200016905/5).

**Table:** Characteristics of persons with and without Omicron BA.1/BA.2 breakthrough infection who received COVID-19 vaccines and booster doses, Denmark*

Characteristics	Vaccines and booster	Vaccines, booster, and Omicron breakthrough
Total no. persons	24	12
Sex, no. (%)		
F	17 (70.8)	8 (66.6)
M	7 (29.2)	4 (33.4)
Median age, y (IQR)	41 (32–51)	47 (40–50)
Median no. days (IQR) between vaccination, infection, and sampling		
Between first and second dose	29 (27–36)	28 (26–34)
Between second and third dose	152 (42–199)	200 (159–228)
Between third dose and sampling	29 (23–34)	90 (77–143)
Between third dose and infection	NA	55 (31–109)
Between infection and sampling	NA	33 (24–38)

*Participants received 3 doses of BNT162b2 (Pfizer-BioNTech, https://www.pfizer.com), 2 initial vaccines and a booster dose. IQR, interquartile range; NA, not applicable

We found serum from the vaccinated cohort neutralized BA.5 with a median PRNT_90_ titer of 20 (IQR 10–40), whereas serum from the convalescent cohort neutralized BA.5 with a median PRNT_90_ titer of 160 (IQR 160–160). Wilcoxon rank-sum test demonstrated this 8-fold increase in neutralization after Omicron infection was statistically significant (p<0.0001). Also, the convalescent cohort neutralized SARS-CoV-2 Delta and Omicron BA.1/BA.2 strains at statistically significantly higher levels than did the vaccinated cohort (Figure, panel A). In vaccinated and convalescent cohorts, BA.5 was neutralized at median titers of 2–8 times lower than for the other virus strains (Figure, panel A). The median levels of SARS-CoV-2 spike-specific antibodies differed between cohorts; the level in the vaccinated cohort was 5,535 (IQR 1,440–8,090) binding antibody units/mL and in the convalescent cohort was 5,675 (IQR 4,970–7,730) binding antibody units/mL, but this difference was not statistically significant by Wilcoxon rank-sum test (p = 0.6505). Moreover, we noted a clear correlation between the levels of spike antibodies and PRNT_90_ titers in the vaccinated cohort (Spearman r_s_ = 0.6624; p = 0.0004) but not in the convalescent cohort (Spearman r_s_ = −0.2048, p = 0.5531) (Figure, panel B).

**Figure:**
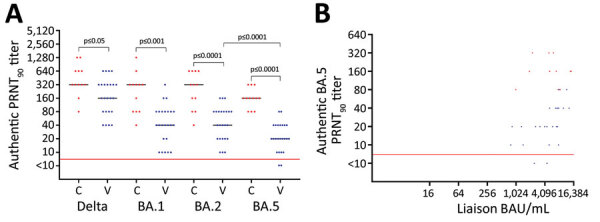
Neutralization of SARS-CoV-2 variants among vaccine-boosted persons with and without prior Omicron BA.1/BA.2 infections, Denmark. A) PRNT_90_ titers against SARS-CoV-2 Delta variant and Omicron variants BA.1, BA.2, and BA.5. B) Correlation between the levels of spike antibodies and PRNT_90_ titers. Participants received 3 doses of BNT162b2 (Pfizer-BioNTech, https://www.pfizer.com), 2 initial vaccines and a booster dose. We analyzed titers for 24 vaccinated participants (blue dots) who received 3 BNT162b2 doses only and 12 convalescent participants (red dots) who received 3 vaccine doses and had Omicron BA.1/BA.2 infection. For statistical analysis, a Kruskal-Wallis test was applied initially to account for the multiple comparisons problem. Subsequently, unpaired PRNT_90_ titers were compared with the Wilcoxon rank-sum test, whereas paired PRNT_90_ titers were compared with the Wilcoxon sign rank test. Red horizontal lines indicate neutralization threshold; horizontal bars indicate median neutralization titer for each SARS-CoV-2 strain. BAU, binding antibody units; C, convalescent participant; PRNT_90_, plaque reduction neutralization tests with plaque reduction >90%; V, vaccinated participant.

Results from published in vitro neutralization studies indicate previous BA.1/BA.2 infection does not confer noticeable humoral protection against BA.5 (*3*–*6*,*9*). We analyzed 2 small but highly matched cohorts that were similar in demography, blood sampling time points, and antibody levels. Our results showed that infection during the spring Omicron BA.1/BA.2 wave greatly strengthened BA.5 neutralization capacity among persons receiving 3-dose vaccine regimens. This result agrees with another recent epidemiologic study that showed prior Omicron infection was highly protective against BA.5 (*7*). As in that study, we collected blood samples from citizens of Denmark during January–April 2022. During that timeframe, BA.2 accounted for 86% of SARS-CoV-2–positive tests in Denmark (https://www.covid19genomics.dk/statistics). BA.5 is closely related to BA.2 (*5*); thus, the BA.5 protection that we and others observe might be because BA.2 was predominant among convalescent patient cohorts, contrary to previous studies that analyzed BA.1 convalescent serum samples (*3*–*5*,*9*).

In this study, Omicron BA.1/BA.2 infection appeared to reduce susceptibility to newer Omicron subvariants such as BA.5 among persons who receive 3-dose BNT162b2 vaccine regimens. Together with the recently reported slower waning of Omicron infection-induced immunity (*10*), this finding suggests that previous Omicron infection confers an appreciable additional degree of humoral protection. 
